# Fostering Social Determinants of Health Transdisciplinary Research: The Collaborative Research Center for American Indian Health

**DOI:** 10.3390/ijerph13010024

**Published:** 2015-12-22

**Authors:** Amy J. Elliott, Emily R. White Hat, Jyoti Angal, Victoria Grey Owl, Susan E. Puumala, DenYelle Baete Kenyon

**Affiliations:** Center for Health Outcomes and Prevention Research, Sanford Research, Department of Pediatrics and Ob-Gyn, Sanford School of Medicine, University of South Dakota, Sioux Falls, SD 57105, USA; emily.whitehat@sanfordhealth.org (E.R.W.H.); jyoti.angal@sanfordhealth.org (J.A.); victoria.greyowl@sanfordhealth.org (V.G.O.); susan.puumala@sanfordhealth.org (S.E.P.); denyelle.kenyon@sanfordhealth.org (D.B.K.)

**Keywords:** American Indian, transdisciplinary, social determinants of health

## Abstract

The Collaborative Research Center for American Indian Health (CRCAIH) was established in September 2012 as a unifying structure to bring together tribal communities and health researchers across South Dakota, North Dakota and Minnesota to address American Indian/Alaska Native (AI/AN) health disparities. CRCAIH is based on the core values of transdisciplinary research, sustainability and tribal sovereignty. All CRCAIH resources and activities revolve around the central aim of assisting tribes with establishing and advancing their own research infrastructures and agendas, as well as increasing AI/AN health research. CRCAIH is comprised of three divisions (administrative; community engagement and innovation; research projects), three technical cores (culture, science and bioethics; regulatory knowledge; and methodology), six tribal partners and supports numerous multi-year and one-year pilot research projects. Under the ultimate goal of improving health for AI/AN, this paper describes the overarching vision and structure of CRCAIH, highlighting lessons learned in the first three years.

## 1. Introduction

Although public health and medical interventions have improved the overall health and life expectancy in the general United States (U.S.) population; racial and ethnic minorities continue to experience disproportionately higher rates of disease and death [1,2,3,4,5]. Poverty, lower education levels, crime, and greater exposure to environmental hazards all contribute to the gradual development and worsening of health status in many minority communities [3,5,6]. The American Indian/Alaska Native (AI/AN) population comprises less than 2% of the U.S. population, yet they bear the greatest burden of many health risk factors and chronic diseases [7,8,9]. The rates of numerous health outcomes reflect the tremendous disparities in AI/AN populations (see Table 1). For example, AI/ANs have an infant mortality rate 132.8% higher than the general population and a 210.8% higher diabetes rate [10,11]. Although there are several ongoing research and service efforts happening at tribal, academic, and health care system levels, projects often occur in isolation. There was a need to bring together these different entities to support collaborative research efforts to address health disparities in AI/AN populations. In September 2012, the Collaborative Research Center for American Indian Health (CRCAIH) was formed at Sanford Research under the Center for Health Outcomes and Prevention Research through $13.5 million in funding from the National Institutes for Minority Health and Health Disparities (NIMHD) to bring together tribal communities and health researchers to address the significant health disparities experienced by AI/AN in South Dakota (SD), North Dakota (ND), and Minnesota (MN). Sanford Research is a non-profit research organization and is part of Sanford Health, an integrated rural health system headquartered in the Dakotas. While Sanford Research is the primary institution of the grant award, CRCAIH is a regional center and this intent is carried throughout the mission, partners and budget. In the last fiscal year, 69.2% of the awarded direct funds went out to the partnering organizations and research projects. Additional funding of CRCAIH’s efforts have been given by Sanford Research in the form of personnel support and incorporation of Sanford resources (e.g., grants office). Additional funds (e.g., Bush Foundation Community Innovation Award) have been received by tribal partner organizations to support the building of their research infrastructures.

**Figure 1:**
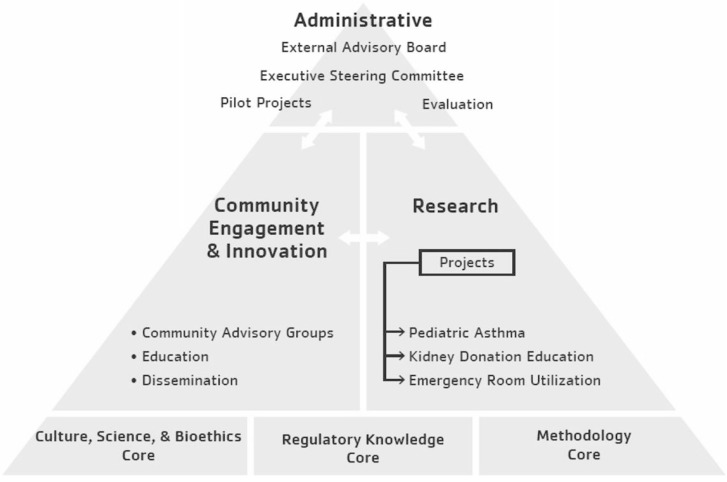
Collaborative Research Center for American Indian Health Organizational Structure.

Research has a troubled history that has resulted in mistrust and skepticism about the role and ability of research to improve AI/AN health. CRCAIH is based on the belief that for AI/AN health research to make a difference, three core values are necessary. These core values are respect for tribal sovereignty, transdisciplinary research, and sustainability. This is being accomplished through a multipronged approach including strengthening tribal research infrastructures, development of core resources, and support of long-term and pilot research projects. The purpose of this paper is to describe the overarching vision and structure of CRCAIH (see Figure 1), after the first three years of operations with tangible lessons learned, outcomes, and implications for others who may be interested in starting similar initiatives.

**Table 1:** American Indian/Alaska Native Health Outcomes.

Health Outcome	US General Population	Overall NHW	Overall AI/AN	SD Overall	SD NHW	SD AI/AN
Infant Mortality—2013 (rate per 1000 live births) [11]	6.01	5.06	8.07	6.96	5.70	11.47
Cancer—2012 (rate per 100,00) [12]	432.3	432.2 ^1^	262.3	426.6	427.2 ^1^	469.8
Diabetes—2012 ^2^ (%) [10]	9.2%	7.9%	17.9%	N/A	N/A	N/A
Heart Disease Mortality 2011–2013 (rate per 100,00) [13]	171.6	173.9	157.2	153.1	150.1	218.4
Heart Disease Incidence—2012 ^2^ (%) [10]	10.8	10.9	12.5	N/A	N/A	N/A

^1^ Includes Hispanic, Non-Hispanic White (NHW); ^2^ age-adjusted percentages, 2012; US—United States; SD—South Dakota; AI/AN—American Indian/Alaska Native.

## 2. Collaborative Research Center for American Indian Health

CRCAIH provides a unified structure across a vast geographic area that brings together tribal communities and researchers with varied backgrounds to address AI/AN health disparities. CRCAIH includes community leaders and seasoned researchers with long histories of successful and mutually beneficial collaborations with Tribal Nations. The partners represent AI/AN Tribal Nations, communities, and organizations, academic institutions, and health care entities (see Table 2). CRCAIH provides a communication and infrastructure platform for all of these entities to discuss individual efforts, build upon the experiences and accomplishments of others and find new collaborative opportunities.

**Table 2:** Collaborative Research Center for American Indian Health Partners.

Tribal Nations and American Indian/Alaska Native Organizations	Research Institutions	Health-Care Organizations
Cankdeska Cikana Community College/Spirit Lake Nation	Sanford Research	Sanford Health
Fond du Lac Band of Lake Superior Chippewa	North Dakota State University	Children’s Hospitals and Clinics of Minnesota
Missouri Breaks, Inc.	South Dakota State University	Rapid City Regional Health
Oglala Sioux Tribe	University of North Dakota	
Rosebud Sioux Tribe	University of South Dakota	
Sisseton Wahpeton Oyate		
Turtle Mountain Band of Chippewa Indians/Tribal Nations Research Group		

### 2.1. Historical Background of CRCAIH

The partners that comprise CRCAIH have long-standing histories of research collaborations based upon Community Based Participatory Research (CBPR) and Tribal Participatory Research (TPR) principles. Both CBPR and TPR embrace the underlying tenets of community involvement at all stages of the research process to create equal partnerships between traditionally trained researchers and community members. All equitable partnerships require sharing power, resources, credit, and results, as well as a reciprocal appreciation of each partner’s knowledge and skills at every stage of the project. These stages include problem definition/issue selection, research design, conducting research, interpreting results, and determining how results should be used for action [14,15]. The Principal Investigator has been partnering with AI/AN tribes on large, complex research studies on maternal child health that include biospecimen collection for 10 years prior to the initiation of CRCAIH. She experienced firsthand the benefits of working with a tribal Research Review Board to better meet the cultural needs of AI/AN research participants.

Guided by the literature on CBPR and TPR, CRCAIH is based on the foundation that AI/AN communities must be more than mere passive participants in pre-developed research protocols [16]. AI/AN communities are sovereign nations with the inherent rights to govern, provide leadership and involvement in all aspects of the research process, including the authority to dictate research agendas and implement solutions [17]. Through our previous research partnerships with tribal nations in the region, often this need for tribally-driven research and greater focus on research in AI/AN health arose, which was the provocation for developing the aims of CRCAIH.

### 2.2. Aims of CRCAIH

One of the primary aims of CRCAIH is to build tribal research infrastructure. Each year, tribes located in SD, ND, and MN are invited to apply and one new tribal partner is added. Each partnering tribe has different existing resources for research and is guided by distinctive visions and goals. Tribes have elected to develop a research review board, tribal research policies/codes, or support new research projects. To meet these goals, CRCAIH provides funding to the tribe for a full-time Tribal Liaison, IRB software, travel, and supplies. Crucial for full community engagement, the Tribal Liaisons are tribal employees, who know their communities and are the local face of CRCAIH. The Tribal Liaisons make connections with community stakeholders, serve as a direct communication link with tribal leadership, facilitate data collection and work on tribal-specific outreach campaigns to promote health research. The work of the Trial Liaisons directly connects with the vision each tribe has for developing their research infrastructures.

The second primary aim of CRCAIH is to increase the amount of transdisciplinary research in AI/AN health. In looking at the literature on AI/AN health outcomes, because there was no clear singular gap in the literature in one health area (e.g., cancer), therefore, the focus of CRCAIH was designed to be broad in scope so the research could support the different needs of each tribal community. As a Transdisciplinary Collaborative Research Center, CRCAIH strives to extend results and impact beyond a single discipline. Transdisciplinary research is becoming an increasingly effective choice for addressing complex public health problems; such as chronic disease prevention and management as these issues have not been successfully addressed by a single discipline approach [18,19,20,21,22,23,24]. CRCAIH is comprised of experts in clinical psychology, counseling, epidemiology, family studies and human development, nursing, medicine, nutrition, public health, sociology, AI/AN culture, and law. The use of transdisciplinary research promotes a reorientation of the theoretical, conceptual, and methodological concepts of each research discipline in the collaboration, creating a novel way to expand knowledge beyond the silo or box of specific disciplines [25]. An important transdisciplinary perspective was added by the Culture, Science and Bioethics Core, highlighting how Indigenous and Western worldviews approach research differently and aspects to consider when building collaborative research relationships.

### 2.3. CRCAIH Divisions and Core Resources

A critical component of CRCAIH, that supports building tribal infrastructures and the conduct of high quality transdisciplinary research, is the availability of research resources. As detailed below, CRCAIH includes three divisions that incorporate administrative responsibilities, community engagement, and research functions as well as three technical cores that provide specialized expertise to all partners (see Figure 1). Each CRCAIH division and core provides training and capacity building services to our tribal partners and research projects. Trainings are often based on specific requests and may be in various formats, including in-person presentations, interactive webinars, hands-on workshops, and web-based training modules. Trainings not only help build CRCAIH partners’ research capacity, but also reach a broader audience of those working in Indian Country.

The Administrative Division is the managerial oversight of activities and communications within and external to CRCAIH. This includes organizing fiscal and contractual documents (dedicated fiscal support is available for partnering entities unfamiliar with managing research grants), development of policies and procedures for CRCAIH activities, leads website development and communicates updates. Internal evaluation of CRCAIH is housed within the Administrative Division, and encompasses strategic planning, review and assessment of division and core resources and outcomes, evaluation support services, and overall guidance and measurement of activities using a developmental evaluation approach [26]. Through use of a developmental evaluation approach the project evaluator is able to make recommendations to promote improvement in service delivery and outcomes throughout the grant period rather than at the end of the grant [26]. This approach has allowed the administrative team to make changes necessary in real-time to accomplish the overall outcomes of the grant.

Key components of the Administrative Division include: an Executive Steering Committee; an External Advisory Board; an Executive Steering Committee; and subcommittees—tribal partner selection, pilot grants program and annual summit. The Executive Steering Committee meets two times a year and includes approximately 40 members with membership from participating tribes, federal funders, and researchers in the region and serves as a communication and dissemination tool for CRCAIH collaborators. The External Advisory Board grew from the Executive Steering Committee to assist in the leadership, advisement, and strategic planning of CRCAIH activities. The External Advisory Board meets three times a year and includes the principal investigator, project director, project evaluator, federal funders, and nationally renowned researchers with expertise in AI/AN research.

#### 2.3.1. Tribal Partner Selection

New tribal partners are chosen each year through an application process. The application process was created because the number of tribes expressing interest in becoming a CRCAIH partner exceeded available resources. This application process was designed to identify the tribes that would make the greatest use of the limited resources to develop their research infrastructures. Each year, CRCAIH starts the application process by reaching out to all tribal governments, tribal health departments, and tribal colleges and universities in the region and invites them to apply. Next, potential tribal partners write a letter expressing interest in building their research infrastructures and how CRCAIH can assist by answering three questions: (1) Why is your tribe interested in growing your research infrastructure; (2) How does this opportunity help further existing tribal initiatives and activities in health research; and (3) What challenges would you anticipate in accomplishing the goals of CRCAIH. Letters are reviewed by the tribal partner selection committee and initial phone calls are made to all applicants by the principal investigator and project evaluator. The top two to four tribes are selected for site visits and follow-up meetings to assess fit (e.g., leadership/community support, potential for research infrastructure growth) with CRCAIH’s overall goals. Three to four CRCAIH committee members attend the site visits, with at least two of the members attending all site visits for consistency in the process. After each visit, committee members fill out the CRCAIH Tribal Selection Criteria form (see Table 3) that includes the following information: (1) Location; (2) Leadership support; (3) Community; and (4) Capacity/Infrastructure. Applicants are ranked via scores from the criteria sheet and the committee meets to review and make a final decision. Key factors that led to a tribe selected include support from the tribal leadership: elected officials and other tribal program directors present during on-site visits; tribal resolutions passed in support of the proposed partnership; and clear indication of supervisor and office space for the tribal liaison. In our experience, the visible display of support from leadership and community members creates the foundation for a successful partnership. This foundation is critical both for advancing their tribes goals and sustaining their infrastructure after the grant is complete.

#### 2.3.2. Pilot Grants Program (PGP)

CRCAIH pilot projects are awarded to promising transdisciplinary investigative teams, with research projects focused on social determinants of health that address significant health disparities experienced by AI/AN in SD, ND, and MN. The duration of each pilot grant is one year with a potential additional carry-over funding year. Each year, $250,000 to $350,000 has been invested in the CRCAIH pilot grants program. The pilot grants subcommittee leads the writing and disbursement of a Request for Applications (RFA) in collaboration with the institutional grants management office. In year one, special attention was paid in developing the application materials to make the forms accessible to junior researchers, while incorporating the information needed for NIMHD review. The proposals are reviewed by an external group of experienced researchers with minority health research backgrounds that live outside the region. The pilot grant review criteria focus on significance, scientific approach, innovation/potential for future funding, investigators/environment, and demonstration of collaborative relationships (letters of support from tribal nations and other partners are required). After the applications are scored and ranked by the external reviewers, the subcommittee selects the final projects, which then go to NIMHD for final approval. Pilot grantees (see Table 4) update CRCAIH through a quarterly reporting mechanism and dissemination of research through presentations, project-generated resources, and manuscripts.

#### 2.3.3. Annual Summit

The annual summit is a critical way for CRCAIH to bring partners together from across this vast region to discuss current and future research in AI/AN health. It serves as a forum for national and regional speakers, trainings, workshops, panel discussions, and CRCAIH researchers. The annual summit serves an important role in dissemination of research findings, highlighting current and past year activities, and moving the AI/AN health research field forward.

The Community Engagement and Innovation Division (CEID) provides the link within CRCAIH to (1) to identify health research priorities with each tribal partner and community members through community engagement; (2) provide information back to the Executive Steering Committee on community health research priorities, which are incorporated into the annual pilot grant RFA; and (3) disseminate information back to communities on the CRCAIH activities and opportunities. Community input and feedback are fundamental to creating the infrastructure necessary for CBPR and TPR; including processing and monitoring requests based on each community’s health research agenda. Community engagement is facilitated through various types of community groups—community advisory boards (CABs), community conferences, forums, and/or working groups to identify community health research priorities, research and training needs, and effective community outreach campaigns.

The Research Division contains three large-scale research projects that utilize varied scientific methodologies and are designed to answer questions raised as priority areas for regional AI/AN communities. The research projects and the pilot projects, serve to demonstrate high quality research and model effective collaborations. The three larger multi-year projects focus on pediatric asthma, kidney donation education, and pediatric emergency department utilization; and all of the projects have an intervention component.

The Culture, Science, and Bioethics Core works with tribes and researchers to provide a culturally-informed approach to research. This core works to find a common language and improve communications between tribes and health researchers. Relationships between the community and the researcher are crucial to successfully conducting meaningful research. Existing relationships can be enhanced and new relationships established, along with the other technical cores of CRCAIH.

The Regulatory Knowledge Core works with tribes and researchers to facilitate appropriate research methods in the modern regulatory framework of protections for human subjects. The Regulatory Knowledge Core helps meet an emergent need among tribal communities to establish and self-govern research on their lands. The Regulatory Knowledge Core provides regulatory support for all CRCAIH projects, including quality control and consultation. These key activities were derived from extensive community input and are intended to bridge significant gaps in the present health research infrastructures. The Regulatory Knowledge Core works with tribal partners to build tribal research capacity through the development of systematic research review processes, including the establishment of community research review boards.

Many tribes are looking to establish procedures that streamline their research review process to ensure consistent and efficient review of research protocols. As CRCAIH extends across multiple states and communities, it brings together entities with varying levels of existing regulatory capabilities. Academic institutions and health systems in the region have well-established research programs, as do some of the participating tribal entities. Finding areas for collaboration and partnership in regulation of research activities helps optimize limited resources. Importantly, these activities build research capacity by fostering partnerships between stakeholders and facilitating exchange of regulatory knowledge.

**Table 3:** Tribal Selection Criteria Scores for Tribes Selected for Site Visits (2013–2015).

	2013			2014				2015		
Tribe	Tribe A *	Tribe B *	Tribe C	Tribe D *	Tribe E	Tribe F *	Tribe G	Tribe H *	Tribe I	Overall Average
**Criteria**										
Location (possible 10 points)	7	8	3	9	6	9	4	8	7	7
Leadership Support (possible 30 points)	24	28	9	27	19	26	16	27	18	22
Community Support (possible 30 points)	23	27	9	21	17	10	14	20	16	17
Capacity/Infrastructure (possible 30 points)	21	28	8	24	10	26	15	19	14	18
Additional Considerations (possible 10 points)	7	9	4	7	7	2	2	0	0	4
**TOTAL**	82	100	37	89	55	90	50	74	55	70

* designates Tribes that were selected as new Tribal Partners.

**Table 4:** Pilot Grant Program Awardees by Year, Institution, and Project Title.

Principal Investigator	Institution	Project Title
**Year 1**		
John Gonzalez, PhD	University of Minnesota Medical School, Duluth	Is my healthcare making me sick? Microaggressions in American Indian healthcare
Jessica D. Hanson, PhD	Sanford Research	Reliability and validity in a prevention program for American Indian women
Alicia Mousseau, PhD	Little Wound School, Oglala Sioux Tribe	Using mindfulness to reduce risky behaviors among American Indian youth
Soonhee Roh, PhD	University of South Dakota	Determinants of care and life quality in American Indian women with cervical cancer
H. Bruce Vogt, MD, FAAP and Jay Memmott, PhD, MSW	University of South Dakota	Assessing the impact of lay patient advocate training in tribal communities
**Year 2**		
Emily Griese, PhD	Sanford Research	Impact of residential treatment on American Indian maternal-child health outcomes
MaryLou Mylant, PhD	South Dakota State University	American Indian pilot study on caregiving attachment and health of young children
Daniel Petereit, M.D	Rapid City Regional Hospital	Walking forward American Indian survivorship physical activity pilot
Heather Peters, PhD	University of Minnesota-Morris	Culturally based curriculum, wicozani and suicidal ideation in Dakota youth
Ursula Running Bear, PhD	University of Colorado-Denver	Multilevel context of health-related quality of life in northern plains tribes
**Year 3**		
Sara DeCoteau, BA and Bonny Specker, PhD	Sisseton-Wahpeton Oyate of the Lake Traverse Reservation and South Dakota State University	Pregnancy Health Survey for Parents of Newborns
Amanda Fretts, PhD	University of Washington	Healthy Food Healthy Families Feasibility Study
Tai Mendenhall, PhD and Kathy Denman-Wilke, MEd	University of Minnesota and St. Paul Area Council of Churches, Department of Indian Work	East-Metro American Indian Diabetes Initiative: An Evaluation of Innovative Community-based Programs to Improve the Health of Native Men and Youth

The goals of the Methodology Core are to assist researchers and communities in developing high quality transdisciplinary research and to help build research infrastructure in tribal communities. Research support is provided by the Methodology Core to any investigators working in AI/AN health disparities and includes assistance with research and pilot projects, pilot grant application development, providing advice and guidance for data management support of other tribally-led research projects and research education. The Methodology Core assists in all aspects of research methodology including study design, outcome identification and measurement, data management, statistical analysis, results interpretation and display and writing of statistical sections of grants, reports and manuscripts. For specific project assistance, the Methodology Core works with individual research groups to determine what is needed. Priority is given to our tribal partners, research and pilot grantees and pilot grant applicants.

## 3. Lessons Learned and Outcomes

### 3.1. Evaluation Findings

**Figure 2:**
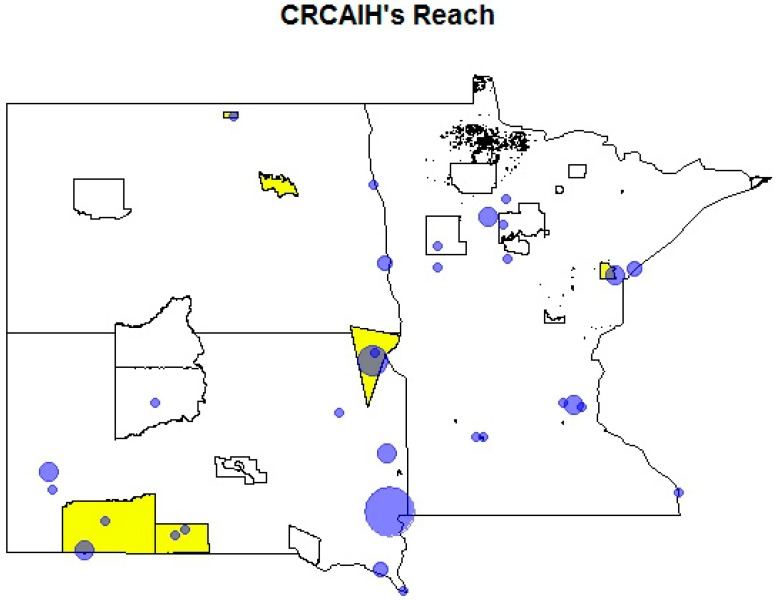
CRCAIH partners and regional collaborations. Bubble size is proportional to the number of collaborations.

Similar to any large scale project, the establishment of CRCAIH has experienced many successes and challenges. The use of developmental evaluation as the method for the internal evaluation of CRCAIH has provided an opportunity for continuous reflection and improvement. We had external evaluators consult throughout the first year to help establish the evaluation framework, and we plan to utilize an external evaluation again in Year 4. Evaluation data has led to the creation of heat maps of the region that focus on reach of trainings, amount of collaborations (e.g., referrals for services), and whether the research and pilot projects are spread across the region (see Figure 2). These heat maps, developed in partnership with the CRCAIH methodology core, show areas of active collaborations and where additional building is needed. The incorporation of evaluation directly in the administrative team has led to issues and areas of concern being addressed in real-time to continuously improve CRCAIH’s outcomes. This has resulted in significant changes, including change in core leadership, establishment of greater fiscal management support, and increased efforts to develop relationships at a national level.

In Year 2, the establishment of greater fiscal management support was crucial to ensure that partners had processes in place to establish clear and concise scope of work documents outlining deliverables, ensure timely reimbursement, and to expend the subaward in its entirety to accomplish the project goals. Unfortunately, despite the increased efforts for fiscal support, CRCAIH did end a subcontract with one tribal partner based on fiscal infrastructure challenges.

Evaluation of the Pilot Grants Program has been vital in assessing the application structure/process, core services offered, and outreach. Each year surveys are conducted with the interested pilot grant applicants, pilot grant reviewers, awarded pilot grantees at the one year completion and annual follow-up. Survey feedback has led to an earlier release of the RFA increasing the amount of time applicants had for application preparation from eight to fourteen weeks, revising review criteria to include a category on collaborative relationships, identifying trainings to help grantees develop competitive applications (e.g., sample size calculations, budget preparation); and identification of tribally-driven health priorities in the region.

A Pilot Grant Program Seminar Series was implemented in year 3, providing an opportunity for pilot grantees to provide a one-hour presentation of the research findings from their funded study to an in person and live streamed audience. The seminar series was developed as an avenue for dissemination and continued investment and mentoring from the CRCAIH divisions and cores. The presentations are archived on the CRCAIH website (www.crcaih.org).

### 3.2. Tribal Partner and Community Impact

The Tribal Partner Selection Process has been successful and reflects the interest of tribal nations in the region to engage in developing research infrastructure and capacity (see inset 1 for case study). As we have been implementing the application model with tribal partners, particularly the past 2 years, we have heard from increasing number of tribes interested in being involved with CRCAIH. In years 2, 3, and 4 we had 3, 7, and 6 numbers of applicants respectively, which includes some repeat applicants. All but 8 of the 23 tribes in the region have either been partners or formally applied to be a CRCAIH partner, and this does not include informal discussions and inquiries. We have also worked to communicate that CRCAIH’s capacity building resources are available to not just tribal partners, but to other tribes and researchers involved in health research with AI/AN populations in ND, SD, and MN.

Inset 1: The Turtle Mountain Band of Chippewa Indians (TMBCI) was selected as a CRCAIH tribal partner in 2013. With help from the CRCAIH infrastructure investment, they have created Tribal Nations Research Group (TNRG), a non-profit dedicated to improving the quality of life for all Turtle Mountain Band of Chippewa Indians (TMBCI) tribal members through culturally-competent, custom-fit research (www.tnrg.org). Notable accomplishments include passing a tribal Research Protection Act and establishing a Research Review Board.

Because of the large geographic area the three states cover, the Annual Summit is hosted in a different location each year to provide an opportunity for those to travel to the summit closer to their location. The summit has provided opportunities for personnel from all components of CRCAIH to network in person and share lessons learned, as well as bring national experts in AI/AN health research to the region to share their knowledge and learn first-hand about AI/AN health conditions in the region.

Through an NIH R13 funding mechanism, the Community Engagement and Innovation Division (CEID) supported two tribal partners in hosting community research conferences—Cheyenne River Sioux Tribe and Missouri Breaks, Inc., hosted the *Researching, Restoring and Rebuilding Our Oyate for a Longer Life* and the Oglala Sioux Tribe hosted the *No Longer Dreaming*—*Your Role in Native American Research*. These community conferences provided an opportunity for tribal member researchers from each tribe to present their research back to their community in a larger forum.

Outreach and communication is accomplished through dissemination of trainings, the CRCAIH website www.crcaih.org, and social media campaigns. This is carried out through a multi-faceted approach which is comprised of (1) web-based technology, including a dedicated website, social media and virtual events; (2) community-based venues, including local newspapers, television and radio stations; and (3) traditional community communication methods, including presentations at individual district health events. The distance and isolation of some communities requires a variety of methods and strategies and each tribe stylizes their campaign to best fit their unique needs. The intended outcome of these outreach measures is increased knowledge and comfort with research, as well as increased empowerment to make a fully-informed decision to engage (or not engage) in research as a tribe or as an individual participant. The contribution of each community is a key measure of CRCAIH’s success.

Many tribes are looking to establish procedures that streamline their research review process to ensure consistent and efficient review of research protocols. One way tribes are accomplishing this is through an electronic review system (see inset 2 for case study). These systematic review processes include management of approved protocols, data and resulting publications. In response to specific requests and inquiries from CRCAIH tribal partners the Regulatory Knowledge Core developed the *Collaborative Research Center for American Indian Health Tribal IRB Toolkit* (available on www.crcaih.org). The toolkit is a compilation of draft protocols, sample process and procedures, and references to trainings developed by the core. RKC presented and recorded a Mock IRB meeting, as well as trainings focused in areas to help determine whether a project is research, whether it involves human subjects, and resources such as a glossary of definitions common in human subjects protections. In addition to the trainings and tools that have been developed through the collaboration with the Regulatory Knowledge Core, three of the five tribal partners have developed research codes, with one tribe entering the CRCAIH partnership with a long standing research code and four of the five tribal partners have operationalized a formal regulatory review process.

Inset 2: The Oglala Sioux Tribe Research Review Board (OSTRRB), in existence since 2007, is one of the few Tribal Institutional Review Boards in the Northern Plains region of the United States. In January 2015 the Oglala Sioux Tribe in Pine Ridge, South Dakota, became the first American Indian Tribe in the Northern Plains to utilize an electronic research management system for submissions, review, and tracking of research conducted on Tribal land. Oglala Sioux Tribe is a partner in the Collaborative Research Center for American Indian Health (CRCAIH). This partnership facilitated the purchase of IRB Software and the transition to the electronic research management system.

The Methodology Core has also developed trainings in the topic areas of introduction to research data, research methods, sample size calculations, and using R Commander. This core has also provided assistance in building tribal research infrastructure through development of data management plans, customized trainings, and offering support for internal research projects.

## 4. Conclusions

### 4.1. Sustainability

As one of CRCAIH’s core values, sustainability has been a constant topic of conversation with our tribal partners and researchers. In addition to the yearly scope of work conversations where we discuss long-term goals of building tribal research infrastructure, we have engaged in strategic planning to help clarify how they can maintain and grow their research infrastructures. One example supported through the Regulatory Knowledge Core is setting up a fee-based system for project review to sustain the tribal Research Review Boards. Sustainability is always a focus with our researchers as well, focusing on how their findings and subsequent products such as publications can lead to future projects and procurement of external funding to sustain their research portfolios. CRCAIH is still quite young, but we feel that this early focus on sustainability will help support the longevity of the program.

### 4.2. Concluding Thoughts

In summary, CRCAIH engages leaders from tribal communities, academic/research organizations, and key healthcare systems in the region. Each of these leaders brings unique qualifications, experiences, and resources to CRCAIH that work in partnership to create an environment supportive and encouraging of collaborative, meaningful, rigorous, and high quality transdisciplinary research in AI/AN health disparities. The individuals and organizations that comprise CRCAIH are well-versed on creating solid communication and interactions despite vast geographic distances. Resources and support to directly increase tribal infrastructure for their active participation in transdisciplinary research is integral to creating sustainable resources with the ultimate goal of decreasing AI/AN health disparities.

## References

[1] Kulkarni S.C., Levin-Rector A., Ezzati M., Murray C.J.L. (2011). Falling behind: Life expectancy in U.S. counties from 2000 to 2007 in an international context. Popul. Health Metrics.

[2] U.S. Department of Health and Human Services National Partnership for Action to End Health Disparities Fact Sheet: HHS Announces Plan to Reduce Health Disparities. http://minorityhealth.hhs.gov/npa/templates/content.aspx?lvl=1&lvlid=39&ID=289.

[3] CERD Working Group on Health and Environmental Health (2008). Unequal Health Outcomes in the United States: Racial and Ethnic Disparities in Healthcare Treatment and Access, the Role of Social and Environmental Determinants of Health, and the Responsibility of the State.

[4] Betancourt J.R. (2004). The Institute of Medicine report “Unequal Treatment”: Implications for academic health centers. Mt. Sinai J. Med..

[5] Betancourt J.R., King R. (2003). Unequal treatment: The Institute of Medicine report and its public health implications. Public Health Rep..

[6] Bullard R.D., Mohal P., Saha R., Wright B. (2007). Toxic Wastes and Race at Twenty: 1987--2007. A Report Prepared for the United Church of Christ Justice and Witness Ministries.

[7] Norris T., Vines P.L., Hoeffel E.M. (2012). The American Indian and Alaskan Native Population: 2010.

[8] U.S. Census Bureau State and County Quick Facts: South Dakota. http://quickfacts.census.gov/qfd/states/46000.html.

[9] Jernigan V.B.B., Duran B., Ahn D., Winkleby M. (2010). Changing patterns in health behaviors and risk factors related to cardiovascular disease among American Indians and Alaska natives. Am. J. Public Health.

[10] Blackwell D., Lucas J., Clarke T. (2014). Summary health statistics for U.S. adults: National health interview survey, 2012. Vital Health Stat. Ser. 10 Data Nat. Health Surv..

[11] Mathews M.S.T.J., MacDorman M.F., Thoma M.E., Division of Vital Statistics. Division of Vital Statistics (2015). Infant mortality statistics from the 2013 period linked birth/infant death data set. National Vital Statistics Reports.

[12] State Cancer Profiles. http://www.statecancerprofiles.cancer.gov/incidencerates/index.php?stateFIPS=00&cancer=001&race=00&sex=0&age=001&type=incd&sortVariableName=rate&sortOrder=default#results.

[13] Centers for Disease Control and Prevention Interactive Atlas of Heart Disease and Stroke Tables. http://nccd.cdc.gov/DHDSPAtlas/Reports.aspx.

[14] Fisher P., Ball T. (2002). The Indian family wellness project: An application of the tribal participatory research model. Prev. Sci..

[15] Fisher P., Ball T. (2003). Tribal participatory research: Mechanisms of a collaborative model. Am. J. Community Psychol..

[16] Israel B., Shulz J., Parker E., Becker A., Allen A., Guzman J., Wallerstein M.M.N. (2008). Critical issues in developing and following community based participatory research principles. Community-Based Participatory Research for Health from Process to Outcomes.

[17] Harding A., Harper B., Stone D., O’Neill C., Berger P., Harris S., Donatuto J. (2012). Conducting research with tribal communities: Sovereignty, ethics, and data-sharing issues. Env. Health Perspect..

[18] Aboelela S.W., Larson E., Bakken S., Carrasquillo O., Formicola A., Glied S.A., Haas J., Gebbie K.M. (2007). Defining interdisciplinary research: Conclusions from a critical review of the literature. Health Serv. Res..

[19] Croyl R.T. (2008). The National Cancer Institute’s transdisciplinary centers initiatives and the need for building a science of team science. Am. J. Prev. Med..

[20] Hall K.L., Stokols D., Moser R.P., Taylor B.K., Thornquist M.D., Nebeling L.C., Ehret C.C., Barnett M.J., McTiernan A., Berger N.A. (2008). The collaboration readiness of transdisciplinary research teams and centers: Findings from the National Cancer Institute’s TREC Year-One evaluation study. Am. J. Prev. Med..

[21] Harper G., Neubauer L., Bangi A.K., Francisco V.T. (2008). Transdisciplinary research and evaluation for community health initiatives. Health Promot. Pract..

[22] Kimer B.K., Abrams D.B. (2012). Present and future horizons for transdisciplinary research. Am. J. Prev. Med..

[23] Mabry P.L., Olster D.H., Morgan G.D., Abrams D.B. (2008). Interdisciplinary and systems science to improve population health: A view from the NIH office of behavioral and social sciences research. Am. J. Prev. Med..

[24] EPSCoR/IDeA Foundation Eligible Jurisdictions and Recent Achievements. http://www.epscorideafoundation.org/about/jurisdictions.

[25] Gray B. (2008). Enhancing transdisciplinary research through collaborative leadership. Am. J. Prev. Med..

[26] Patton M. (2011). Developmental Evaluation: Applying Complex Concepts to Enhance Innovation and Use.

